# Commentary: Randomized Phase II Study of Duligotuzumab (MEHD7945A) vs. Cetuximab in Squamous Cell Carcinoma of the Head and Neck (MEHGAN Study)

**DOI:** 10.3389/fonc.2017.00031

**Published:** 2017-03-13

**Authors:** Nabil F. Saba

**Affiliations:** ^1^Hematology and Medical Oncology, Winship Cancer Institute of Emory University, Atlanta, GA, USA

**Keywords:** squamous cell carcinoma of the head and neck, cetuximab, EGFR, HER3 inhibitors, Nrg1

We read with great interest the report by Fayette et al. summarizing the results of a phase II study of duligotuzumab (MEHD7945A) versus cetuximab in squamous cell carcinoma of the head and neck (SCCHN) and congratulate the authors on completing and publishing this phase II multinational trial (1).

This trial compared cetuximab to a dual HER3 and EGFR targeting IgG1 antibody (duligotuzumab) in patients with recurrent or metastatic SCCHN who have progressed on at least one platinum-based regimen. Although we agree that the results do not signal a clinical benefit for the dual antibody in comparison to cetuximab, we have reservations about the authors’ conclusion that the results provide definitive clinical evidence refuting the hypothesis that the dual blockade of HER3 and EGFR may have clinical merit, especially for patients whose tumors express NRG1.

We would like to point out, in that regard, that preclinical evidence overwhelmingly suggests that HER3 and its ligand function as compensatory mechanisms to EGFR blockade and are hence linked to decreased sensitivity to cetuximab (2, 3). Even though arguably, mechanisms of cetuximab resistance may be at play in cetuximab-naïve patients, it is clear from preclinical studies that HER3 activation is responsible for cetuximab resistance developing under the pressure of EGFR blockade; furthermore, sensitivity to EGFR inhibition can be restored with the inhibition of HER3, further supporting the role of HER3 and its ligand mostly in the acquired rather than innate resistance to EGFR inhibition (2, 3). In light of this, it is legitimate to ask whether a cetuximab-naïve patient population would stand to benefit the most from this the dual and simultaneous EGFR/HER3 blockade. It is worth noting here that one possible reason for the lack of benefit in studies with dual EGFR and HER3 or HER2 inhibitors such as afatinib (4) in SCCHN could well be patient selection. None of these trials focused on patients with the arguably higher likelihood of resistance namely those with prior cetuximab exposure. Furthermore, the conclusion reached in Fayette et al. refuting the role of HER3 and its ligand in cetuximab resistance contradicts a large volume of existing evidence, thereby supporting a role for HER3 in the acquired resistance to EGFR inhibition (2, 3). The authors rightfully point to the substantial body of evidence in that respect. In addition, a higher level of NRG1 mRNA observed in SCCHN compared to other malignancies, as noted by the authors, has also been corroborated by data from TCGA (5), pointing to an important role of HER3 and NRG1 in SCCHN. It is noteworthy, however, that the observed clinical benefit seems to be in the HPV-negative group, in contrast to other analyses indicating no clear difference based on HPV status (5). Finally, despite the robust pharmacokinetic data provided in the analysis, it is fair to ask whether a dual antibody such as duligotuzumab has the same clinical effectiveness as a combination of antibodies to EGFR and HER3 (3, 6). We believe nevertheless that the Fayette et al.’s study further enhances our understanding of pathways of resistance to EGFR and underscores that targeting these pathways is a daunting task in SCCHN, as well other tumors. We believe, however, that successful targeting must be approached carefully, taking various factors into consideration, not the least of which is patient selection. This, in our view, ought to be based on an appropriate biomarker profile as well as exposure to and likely prior failure of EGFR-based therapy. We again commend the authors on their substantial effort in completing this important study.

## Author Contributions

The author confirms being the sole contributor of this work and approved it for publication.

## Conflict of Interest Statement

The author declares that the research was conducted in the absence of any commercial or financial relationships that could be construed as a potential conflict of interest.
